# Improving the effectiveness of mental health care through the use of PROMs and PREMs; the OECD perspective

**DOI:** 10.1192/j.eurpsy.2023.127

**Published:** 2023-07-19

**Authors:** K. De Bienassis

**Affiliations:** OECD, Paris, France

## Abstract

**Abstract:**

Patient-reported measures are a critical tool for improving policy and practice in mental health care. However, to date, the use of patient-reported measures in mental health care is limited to a small number of countries and settings—and there is a pressing need, both within and across countries, to consistently and effectively measure the effects and impact of care for patients who use mental health care services. The PaRIS pilot data collection on mental health included 15 data sources from 12 countries, collected over the course of 2021. While the scope of included data varied, the results demonstrate increased adoption of national and subnational efforts to capture patient-reported information in mental health care systems. Analysis of data collected through the PaRIS mental health pilot documents, in general, positive patient-reported experiences of mental health care. The results also suggest improvement in patient-reported outcomes for those receiving mental health care services.

**Disclosure of Interest:**

None Declared

